# 1421. A 5-year Review of *Mycobacterium abscessus* Susceptibility in a Tertiary Hospital in Thailand

**DOI:** 10.1093/ofid/ofac492.1250

**Published:** 2022-12-15

**Authors:** Pawat Phuensan, Suthidee Petsong, Gompol Suwanpimolkul

**Affiliations:** King Chulalongkorn Memorial Hospital, Krung Thep, Krung Thep, Thailand; Chulalongkorn University, Krung Thep, Krung Thep, Thailand; Chulalongkorn University, Krung Thep, Krung Thep, Thailand

## Abstract

**Background:**

*Mycobacterium abscessus* is a common rapid growing mycobacteria (RGM). It causes chronic nodular or cavitary lung disease in adults with bronchiectasis or cystic fibrosis, skin and soft tissue infection following penetrating injury or an unsterile skin procedure. It was also a pathogen of disseminated infection in patients with anti-interferon-gamma autoantibodies. The 2018 clinical and laboratory standards institute (CLSI) guideline suggested that amikacin, cefoxitin, ciprofloxacin, clarithromycin, doxycycline, imipenem, linezolid, meropenem, moxifloxacin, trimethoprim-sulfamethoxazole, tigecycline and tobramycin should be tested against RGM. We aimed to review the antimicrobial susceptibility of this organism in our hospital.

**Methods:**

We performed a retrospective descriptive review of the minimal inhibitory concentration (MIC) of clinical isolates of *M*. *abscessus* from 2013 to 2018.

**Results:**

We found 267 isolates of M. abscessus. Eighty-four isolates were tested for MICs. Of these, 4 isolates were excluded due to duplication. The remaining 80 isolates were included for analysis. The susceptibility results were as follows.

**Conclusion:**

To our knowledge, this is the largest report of susceptibility pattern of *M*. *abscessus* in Thailand. MICs of cefoxitin and imipenem, which are the recommended intravenous antimicrobials, were high. Most isolates also demonstrated high MICs of doxycycline, linezolid, trimethoprim-sulfamethoxazole, tobramycin, ciprofloxacin and moxifloxacin. In contrast, most of MICs of amikacin and clarithromycin were in susceptible range. These findings may be used to guide the treatment regimen although clinical outcomes of each drug are still to be investigated.

**Disclosures:**

**All Authors**: No reported disclosures.

**Table:** 

Antimicrobial agents (N)	Susceptible (%)	Intermediate (%)	Resistant (%)
Amikacin (80)	86.25	7.50	6.25
Cefoxitin (80)	3.75	31.25	65
Ciprofloxacin (79)	1.27	3.80	94.93
Clarithromycin (80)	97.50	0	2.50
Doxycycline (32)	0	9.38	90.62
Imipenem (80)	0	15.00	85.00
Linezolid (26)	50.00	23.08	26.92
Moxifloxacin (26)	0	7.69	92.31
Trimethoprim-sulfamethoxazole (80)	2.5	0	97.5
Tobramycin (24)	4.17	20.83	75

